# The Health Risk of Cd Released from Low-Cost Jewelry

**DOI:** 10.3390/ijerph14050520

**Published:** 2017-05-12

**Authors:** Miloslav Pouzar, Magdalena Zvolská, Oldřich Jarolím, Lenka Audrlická Vavrušová

**Affiliations:** 1 Institute of Environmental and Chemical Engineering, Faculty of Chemical Technology, University of Pardubice, Studentska 573, 532 10 Pardubice, Czech Republic; Miloslav.Pouzar@upce.cz (M.P.); Lenka.AudrlickaVavrusova@upce.cz (L.A.V.); 2 Center of Materials and Nanotechnologies (CEMNAT), Faculty of Chemical Technology, University of Pardubice, Nam. Cs. Legii 565, 530 02 Pardubice, Czech Republic; 3 Department of Waste Management, Czech Environmental Inspectorate, Na Břehu 267, 190 00 Praha 9, Czech Republic; Oldrich.Jarolim@cizp.cz

**Keywords:** health risk, low-cost jewelry, artificial sweat, cadmium, laser-induced breakdown spectrometry

## Abstract

The composition of the surface layer of 13 low-cost jewelry samples with a high Cd content was analyzed using an energy-dispersive X-ray fluorescence spectrometer (ED XRF). The analyzed jewels were obtained in cooperation with the Czech Environmental Inspectorate. The jewels were leached in two types of artificial sweat (acidic and alkaline) for 7 days. Twenty microliters of the resulting solution was subsequently placed on a paper carrier and analyzed by an LIBS (Laser-Induced Breakdown Spectrometry) spectrometer after drying. The Cd content in the jewelry surface layer detected by using ED XRF ranged from 13.4% to 44.6% (weight per weight—w/w). The samples were subsequently leached in artificial alkaline, and the acidic sweat and leachates were analyzed using laser-induced breakdown spectrometry (LIBS). The amount of released Cd into alkaline sweat ranged from 24.0 to 370 µg Cd per week, respectively 3.23–61.7 µg/cm^2^/week. The amount of released Cd into acidic sweat ranged from 16.4 to 1517 µg Cd per week, respectively 3.53–253 µg/cm^2^/week. The limit of Cd for dermal exposure is not unequivocally determined in the countries of the EU (European Union) or in the U.S. Based on the US EPA (United States Environmental Protection Agency) approach used to establish the reference dose (RfD) for Cd contained in food and information about the bioavailability of Cd after dermal exposure, we assessed our own value of dermal RfD. The value was compared with the theoretical amount of Cd, which can be absorbed into the organism from jewelry in contact with the skin. The calculation was based on the amount of Cd that was released into acidic and alkaline sweat. The highest amount of Cd was released into acidic sweat, which represents 0.1% of dermal RfD and into alkaline sweat, 0.5% of dermal RfD. These results indicate that the analyzed jewelry contains Cd over the limit for composition of jewelry available within the territory of the EU. The determined amount of Cd in analyzed jewelry does not, however, pose a threat in terms of non-carcinogenic toxic effects.

## 1. Introduction

Cadmium is a toxic metal that is easily accumulated in the human body. Even low exposure levels can cause accumulation in human tissues, especially in the kidneys [1]. Chronic cadmium poisoning can inflict renal dysfunction or emphysema, among other afflictions [2]. A number of epidemiological studies have suggested that cadmium is a human carcinogen. According to the EU (European Union) regulation (No. 1272/2008) on classification, labeling, and packaging of substances and mixtures, cadmium is classified as carcinogenic (cat. 1B), mutagenic (cat. 2), toxic for reproduction (cat. 2), acutely toxic (cat. 2), and toxic for the aquatic environment.

The European Commission issued a document on the results of a risk evaluation and on risk reduction strategies for cadmium and cadmium oxide (2008/C 149/03) in 2008. The commission concluded that risk management measures were needed for the protection of consumers because of concerns for genotoxicity and carcinogenicity irrespective of the route of exposure, as the substance was considered a non-threshold carcinogen, arising from wearing (imported) jewelry. As of 2011, cadmium has been restricted in jewelry in EU/EEA (European Union/European Economic Area) countries by the REACH (Registration, Evaluation, Authorisation and Restriction of Chemicals) regulation (No. 1907/2008), Annex XVII, entry 23(10). This restriction was implemented by Commission Regulation No. 494/2011 and limits the concentration of cadmium in the metal parts of jewelry and imitation jewelry articles and hair accessories to a maximum of 0.01% by weight. Jewelry and jewelry-like articles containing cadmium over the limit cannot be placed on the market in EU/EEA countries as of January 2012.

The number of non-compliant jewelry articles on the market is deemed to be extremely high. The Czech Environmental Inspectorate tested, for example, 105 random pieces of jewelry placed on the market in the Czech Republic in 2015. Twenty-three articles contained cadmium levels over the limit. The average cadmium content of those non-compliant articles was 35% w/w (weight per weight). Non-compliant articles were found in 2016 as well, the maximum amount of cadmium found in an article was 91% w/w. It can be concluded that cadmium in jewelry is not present as an unwanted contaminant but is rather deliberately used during the production of jewelry articles. Cadmium is used in all probability in the production of such articles due to its favorable properties. It is easy to utilize, resistant to rust, and relatively cheap. Jewelry articles with cadmium are also abundant in other EU/EEA states. The database of the Rapid Exchange of Information System RAPEX (The Rapid Alert System for non-food dangerous products); EU alert system for unsafe consumer products) listed, for example, 157 notifications from member states regarding cadmium jewelry over the 2012–2016 period [3]. Notifications involving cadmium jewelry amounted to 6.5% of all RAPEX notifications concerning chemical risk in 2016.

Cd content is not only limited in jewelry, but also in other objects of human daily use, such as cosmetics, [4] articles for contact with food [5,6], or children’s toys [7]. Cd content is monitored in textile or plastic in EU countries [8], with the Cd limit for both types of articles being 0.01 wt %. 

In case of skin contact with objects containing Cd, the possibility of dermal exposure and the emergence of various types of irritation have been discussed. Assays for assessing the dermal toxicity of Cd have been described in several publications [9,10,11]. The test of percutaneous absorption of Cd was also performed on human skin samples [12]. The aim was to determine the absorption of Cd as a chloride salt from the aqueous solution through human skin into the plasma. Only 0.5 or 0.6% of the total amount of Cd, which was contained in the aqueous solution, was then absorbed through the skin into the blood plasma. According to the authors, the surface Cd concentration has an influence on the amount of Cd diffused into the skin, but Cd transfer into the plasma is independent from the concentration of Cd applied to the skin.

To determine the amount of released analyte from a solid sample, it is advisable to perform a leaching test. An artificial human sweat was used as a leaching agent for objects that may come into contact with the skin. A number of model solutions with defined content elements, organic compounds, pH, etc. were discussed. Artificial human sweat has been used to dissolve the chemical components of jewelry, textiles, cosmetics, pharmaceuticals, industrial chemicals, and others [13]. Tests of leaching are often used for determining the amount of released Ni from objects that are in contact with the skin. Ni is a significant allergen, which can cause dermatitis and other allergic reactions amongst sensitive individuals. Determination of Ni in sweat extracts of the analyzed objects has been described in several publications [14,15,16,17,18,19].

Non-destructive methods such as X-ray fluorescence spectrometry (XRF) can be used for analysis of the surface composition of the jewelry [20]. In order to assess the amount of Cd released from jewelry into human perspiration and for the assessment of dermal exposure, a leaching test with simulated human sweat can be performed. The most commonly used methods for water solution analysis are AAS (Atomic Absorption Spectrometry), ICP OES (Inductively Coupled Plasma Optical Emission Spectrometry), and ICP MS (Inductively Coupled Plasma Mass Spectrometry). Here, we provide a new perspective on the possibility of leachate analysis and the determination of toxic elements in said leachates using Laser-Induced Breakdown Spectrometry (LIBS). Depositing a small solution volume on a solid support can be a suitable alternative method of analysis of leachates and a new option for the assessment of non-compliant subjects. This method could bring several advantages, such as speed of analysis, the minimization of the consumption of the sample, and the possibility of storing the dried samples and adequate detection limits.

## 2. Materials and Methods 

### 2.1. Samples 

Samples of cheap Cd containing jewelry were obtained from the Czech Environmental Inspectorate. The subject of our interest was a total of 13 pieces of jewelry (3 sets of earrings, 6 pendants, and 1 ring) originating from inspections of three e-shops trading in cheap Chinese goods. Illustrative photos of the analyzed jewelry are shown in Table 1. 

### 2.2. Analysis of Surface Composition 

The surface composition of the samples was analyzed using an Elva X energy-dispersive X-ray fluorescence spectrometer (Elvatech Ltd., Kiev, Ukraine) equipped with a Pd X-ray tube and a thermoelectrically cooled Si-pin detector, PF 550 (MOXTEC, Orem, UT, USA). The power supply of the X-ray tube was operated at 40 kV, and the current was set via the auto-optimization procedure taking into account the optimal loading of the detector in a range of 6000–6500 counts per second (cps). The spectra were integrated for 90 s. Each sample was analyzed at five measuring points evenly spaced on that part of the sample that was supposed to be in contact with the skin. Parts of the sample that usually do not come in direct long-term contact with the skin, such as the solder holding the stone, were omitted from the analysis. Concentrations of elements detected in the samples were calculated by the standard-less module based on the fundamental parameters method. 

### 2.3. Leaching the Samples in an Artificial Sweat

The leaching of jewelry samples was performed with two types of artificial sweat—acidic and alkaline [21]. Acidic sweat was prepared dissolving 0.5 g of l-histidine mono-chloride monohydrate (C_6_H_9_O_2_N_3_·HCl·H_2_O), 5 g of NaCl, and 2.2 g of NaH_2_PO_4_·2H_2_O in 1 L of demineralized water. The pH value was adjusted by 0.1 moL·L^−1^ NaOH to 5.5. To prepare 1 L of alkaline sweat, 0.5 g of C_6_H_9_O_2_N_3_·HCl·H_2_O, 5 g of NaCl and 5 g of Na_2_HPO_4_·12H_2_O was diluted in demineralized water. A solution of NaOH of a concentration of 0.1 moL·L^−1^ was added for a pH adjustment to 8. The volume of artificial sweat for the leaching of the particular jewelry piece was chosen based on the sample surface area, so that 1 mL of the reagent was used for each 1 cm^2^ of the surface. The sample parts, which were not supposed to come into direct contact with the skin (e.g., stones), were not included into the calculated surface. These parts were covered by resistant adhesive tape during leaching to prevent the release of Cd. The leaching procedure lasted 7 days and was performed at 37 °C. The pieces of jewelry were then removed from the leachate, and 20 μL of the solution was spotted onto a circular piece of paper with a diameter of 17 mm and dried under an infrared lamp. 

Leachate samples deposited on the paper carrier were analyzed using the commercially available compact LIBS spectrometer (LEA S500, Solar TII Ltd., Minsk, Belarus). The system consists of a dual pulse Q-switched Nd:YAG (Neodymium-doped Yttrium Aluminum Garnet) laser operating at 1064 nm. A nanosecond laser emitting two collinear pulses of 12 ns was operated at double-pulse mode with an inter-pulse delay of 7 μs. A laser beam with an energy of 110 mJ was focused on the sample surface, where the analytical point with a diameter of 200 μm was ablated. Each sample was analyzed at nine independent analytical points, while every point was ablated by one laser shot. Radiation emitted by arising plasma was led through the entrance slit of 25 μm into a Czerny-Turner monochromator and the spectral window in a range from 205 to 235 nm was recorded by a back-thinned and front-illuminated CCD (Charge-Coupled Device) camera (2048 × 14 pixels). Quantitative analysis of Cd was carried out on an analytical line of 214.441 nm. Separate calibration curves were constructed for acidic and alkaline sweat samples in a range from 0 to 40 mg·L^−1^. Solutions of artificial sweat, spiked with Cd, were used as calibration standards. The precision and accuracy of the LIBS methods was validated comparing LIBS and ICP OES for a set of 5 samples of each sweat type. The mean concentration values measured by both methods were equal, although the LIBS results suffered from a higher standard deviation. The sample volume needed for the analysis was substantially lower, by contrast, in the case of LIBS, and this method also offered the possibility of the long-term storage of liquid samples deposited on the solid carrier.

## 3. Results and Discussion

### 3.1. Surface Analysis

The analyzed jewelry samples revealed a truly variable surface composition. As can be seen in Table 1, the surface layer of all the samples contained Cd (13.4–44.6%), Zn (0.5–39.7%), and Cu (25.8–85.4%). A measurable content of Ag was contained in 11 out of the 12 samples (0.2–1.6%); Sn was detected in 4 samples (0.2–04%), and Ni was detected in 8 samples (0.3–13.8%). The Cd concentrations detected in the jewelry samples using ED XRF significantly exceeded the appropriate limits valid in the EU (0.01%), the USA (0.03%), and Canada (0.013%).

### 3.2. Leaching in Artificial Sweat

After measuring calibration standards, the limit of detection (LOD) for both acid and alkaline artificial sweat solutions was determined. The LOD was determined according to definition 3σ/*s*, where σ is the standard deviation of intensity calculated from 36 repeated measurements of the lowest calibration standard (blank) performed under optimal conditions, and *s* is the slope of the calibration curve. The calculated LOD has a value of 0.08 mg·L^−1^ and 0.06 mg·L^−1^ for acidic and alkaline artificial sweat, respectively. The relative standard deviation (RSD; %) calculated for measuring data of jewelry leachates was in a range from 4.79% to 22.6%. 

One piece of each pair of earrings (1B, 2B, and 3B) was leached in acidic and one piece in alkaline artificial sweat. Data presented in Table 2 reveal that the total amount of cadmium, which was released into the alkaline sweat, constitutes in all cases about 20% of the amount released into the acidic sweat. Apart from the earrings, four pieces of pendants (4A, 6A, 7A, and 9A) were leached in alkaline sweat and two pieces of pendants (5A and 10A) together with one piece of ring in acidic sweat. The total amounts of Cd released into the particular types of artificial sweat are not clearly correlated with the Cd content in the sample surface layer. The correlation coefficients calculated for Cd content in jewelry and in alkaline (*r* = 0.786, *p* = 0.04) or in acidic sweat (*r* = 0.830, *p* = 0.04) were quite high, but these results were strongly biased by the influential point of 1B. After exclusion of the influential 1B point, the correlation coefficients significantly decreased (alkaline sweat *r* = 0.614, *p* = 0.19; acidic sweat *r* = 0.607, *p* = 0.28). The same relations were observed when the correlation coefficients were calculated for Cd content measured by ED XRF and for Cd released from one square centimeter of sample over one week (% Cd w/w vs. µg Cd/cm^2^/week). The correlation coefficients for alkaline (*r* = 0.763, *p* = 0.05) and acidic (*r* = 0.741, *p* = 0.09) sweat were also quite high and similar, but these results were also biased by the influential point of 1B. In this case, the sharp decline in the correlation coefficient was also observed after exclusion of the influential 1B point (alkaline sweat *r* = 0.312, *p* = 0.55; acidic sweat *r* = 0.56, *p* = 0.33). It is apparent that the available set of samples is too small in number to be able to clearly describe the relationship between the surface composition of the sample and the amount of leached cadmium. Streicher porte et al. in their work from 2008 analyzed 21 samples of low cost jewelry containing 1.4–43.9% of Cd on the surface layer. Migration of the toxic metals was tested after sample submersion in 0.07 M HCl (Hydrochloric acid-simulation of gastric acid) for 7 days at 30 °C [20]. The observed correlation between the released Cd in µg/cm^2^/week was low (*r* = 0.49), which is in agreement with our results, if we evaluate the correlation after exclusion of the influential points. 

The obtained sample set contained 3 pairs of earrings. One earring out of the pair was leached in acidic and the other in alkaline artificial sweat. When comparing the results from leachate extracts for pairs of earrings, alkaline artificial sweat provided higher results for dissolved Cd. This is surprising because it is generally assumed that metals are better dissolved in an acidic environment. 

### 3.3. Systemic Non-Carcinogenic Health Risk of Released Cd 

A reference dose (RfD) is the regulatory limit established by the United States Environmental Protection Agency (US EPA) representing the maximum oral dose of a toxic substance, below which no adverse non-carcinogenic health effects should result from a lifetime of exposure. According to the Integrated Risk Information System [22], the calculation of RfD for chronic oral exposure was based on the assumption of increased proteinuria occurring when the Cd content in the renal cortex exceeds the value of 200 µg Cd/g wet tissue. Cd daily intake of 0.352 mg (or 0.005 mg/kg/day for 70-kg adult), which is necessary in order for the concentration of this element in the renal cortex to reach the critical value, was estimated based on the work of Friberg et al. [23]. This work assumed a Cd biological half-life (t_1/2_) of 19 years, an exposure duration of 50 years, and an absorption of 4.5% of Cd contained in the food. US EPA postulated only 2.5% absorption of Cd from the food and consequently established the NOAEL (No-Observed-Adverse-Effect Level) value of 0.01 mg of Cd/kg/day. RfD of 0.001 Cd/kg/day was then obtained dividing NOAEL by the uncertainty factor (UF) of 10. The US EPA document shared at the IRIS (Integrated Risk Information System) database unfortunately does not provide any further information regarding the used toxicokinetic model. When the one-compartment standard first-order elimination model with bolus administration described by Amzal et al. [24] was used, the same value of NOAEL (0.01 mg Cd/kg/day) was obtained for the subsequent set of parameters: a Cd gastrointestinal absorption index equal to 2.5%; a fraction of absorbed Cd transported to the kidney equal to 33%; a ratio of Cd content in the entire kidney and renal cortex of 1.25; a kidney weight of equal to 300 g; and a Cd biological half-life t_1/2_ of 18.3 years. 

The EPA did not establish a limit of a similar meaning as the RfD for dermal exposure. To be able to assess the health risk of Cd released from low-cost jewelry, we performed our own approximation of dermal RfD based on the same toxicokinetic model. The parameters mentioned above were used except for the absorption index, which was set to 0.6%. The calculated value of NOAEL for dermal exposure was 0.042 mg/kg/day. The resulting dermal RfD in this case was also obtained dividing NOAEL by the uncertainty factor UF = 10 with a value of 0.004 mg/kg/day. The dermal RfD estimated in such a way represents a kind of worst-case scenario, in as much as the Cd absorption into the plasma could be lower than the absorption into the renal cortex with published data for absorption into the plasma varying between 0.1% and 0.6% [11]. 

The total amounts of Cd in µg released from a particular piece of jewelry (TRA—Total Released Amount) into acidic or alkaline artificial sweats over one week of leaching are summarized in Table 1. The maximum absorbable daily dose (MADD) of Cd from a particular piece of jewelry was calculated based on the assumed Cd bioavailability of 0.6% and an average human body weight of 70 kg according to the following equation: MADD = 0.006 × TRA/(70 × 7). A factor of 7 in the denominator was used to convert the amount of Cd released over one week of leaching to the daily exposure. The risk characterization ratio (RCR) was then calculated as the hundredfold ratio of MADD and RfD. This factor serves as an estimate as to what percentage of the safe daily dose can be covered by Cd released from the jewelry. The amount of Cd leached into alkaline artificial sweat typically represents about 0.01–0.02% of the safe daily dose, while the maximal RCR for Sample 1B was 0.1%. In the case of acidic artificial sweat, RCR values are higher and more variable (ranging from 0.05% to 0.46%). Although the process of health risk estimation used is extremely simplified, it can be concluded that the evaluated set of Cd containing jewelry do not pose any serious health risk in terms of systemic non-carcinogenic effects.

## 4. Conclusions

A composition of a surface layer of 13 low cost jewelry samples with a high content of Cd was analyzed by ED XRF. These samples were subsequently leached in artificial acidic and alkaline sweat, and the resulting digests were applied onto solid carriers and analyzed by LIBS. The content of Cd in the jewelry surface layer ranged from 13.40% to 44.64% (w/w) with the measured values significantly exceeding permissible limits in the EU or U.S. The results of the analysis suggest that this jewelry should not be available in the countries of the EU. The analysis of the leachates indicates that acidic artificial sweat released an amount of Cd roughly 5-fold higher than that of artificial alkaline sweat. The relationship between the surface composition of the samples and the amount of Cd released into artificial sweat was not clearly demonstrated. The low bioavailability of Cd for dermal exposure, along with the small amounts of Cd released from the surface layer of the jewelry, leads to the conclusion that even the long-term use of these jewels does not constitute major health risks in terms of the biological and toxic effect of Cd. The maximum amount of released Cd from the analyzed jewelry makes up about 0.5% of a safe dose. 

## Figures and Tables

**Table 1:** Surface composition of jewelry from an Energy-Dispersive X-ray Fluorescence Spectrometer (ED-XRF) and parameters of the analyzed samples.

	ED XRF [%, w/w]								
Sample	Cd	Cu	Zn	Sn	Ni	Ag	Area (cm^2^)	Weight (g)	Picture
1B	44.64	35.82	5.55	0.00	13.76	0.23	6.00	5.676	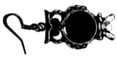
2B	34.52	25.78	39.71	0.00	0.00	0.00	9.00	10.24	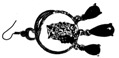
3B	26.59	46.00	25.31	0.20	0.35	1.55	4.00	2.58	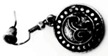
4A	24.96	64.73	8.20	0.21	0.47	1.42	3.00	4.21	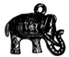
6A	29.13	57.07	7.18	0.00	0.00	6.63	1.00	3.59	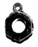
7A	13.69	65.01	20.00	0.00	0.93	0.37	6.25	4.07	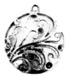
9A	29.11	38.06	32.00	0.00	0.58	0.25	9.00	4.63	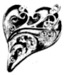
5A	25.73	64.30	9.20	0.00	0.54	0.24	12.50	4.80	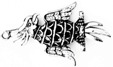
10A	19.50	52.49	26.63	0.39	0.34	0.65	4.00	2.88	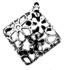
Ring	13.40	85.41	0.54	0.15	0.49	0.00	3.00	7.09	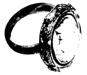

**Table 2:** The total amount of cadmium that was released into the artificial sweat, and calculated values for MADD and RCR.

Sample	Sweat Type	TRA Cd (µg/Week)	TRA Cd * (µg/cm^2^/Week)	MADD (µg/kg/Day)	RCR (%)
1B	Alkaline	370.4	61.73	4.54 × 10^−3^	1.13 × 10^−1^
2B	Alkaline	72.70	8.078	8.90 × 10^−3^	2.23 × 10^−2^
3B	Alkaline	49.01	12.25	6.00 × 10^−4^	1.50 × 10^−2^
4A	Alkaline	29.37	9.789	3.60 × 10^−4^	8.99 × 10^−3^
6A	Alkaline	24.55	24.55	3.01 × 10^−4^	7.52 × 10^−3^
7A	Alkaline	24.00	3.840	2.94 × 10^−4^	7.35 × 10^−3^
9A	Alkaline	29.09	3.232	3.56 × 10^−4^	8.90 × 10^−3^
1B	Acidic	1517	252.9	1.86 × 10^−2^	4.64 × 10^−1^
2B	Acidic	340.5	37.83	4.17 × 10^−3^	1.04 × 10^−1^
3B	Acidic	222.3	55.585	2.72 × 10^−3^	6.81 × 10^−2^
5A	Acidic	44.14	3.532	5.41 × 10^−4^	1.35 × 10^−2^
10A	Acidic	16.36	4.089	2.00 × 10^−4^	5.01 × 10^−3^
Ring	Acidic	154.6	51.53	1.89 × 10^−3^	4.73 × 10^−2^

TRA Cd—total released amount of Cd per 1 week; TRA Cd *—total released amount of Cd per 1 week from 1 cm^2^ of jewelry; MADD—maximum absorbable daily dose; RCR—risk characterization ratio.
